# Maternal–infant interaction quality is associated with child *NR3C1* CpG site methylation at 7 years of age

**DOI:** 10.1002/ajhb.23876

**Published:** 2023-02-13

**Authors:** Elizabeth A. Holdsworth, Lawrence M. Schell, Allison A. Appleton

**Affiliations:** ^1^ Department of Anthropology Washington State University Pullman Washington USA; ^2^ Department of Anthropology University at Albany State University of New York Albany New York USA; ^3^ Department of Epidemiology & Biostatistics University at Albany State University of New York Rensselaer New York USA

## Abstract

**Objective:**

Infancy is both a critical window for hypothalamic–pituitary–adrenal (HPA) axis development, and a sensitive period for social–emotional influences. We hypothesized that the social–emotional quality of maternal–infant interactions are associated with methylation of HPA‐axis gene *NR3C1* later in childhood.

**Methods:**

Using a subsample of 114 mother‐infant pairs from the Avon Longitudinal Study of Parents and Children (ALSPAC), linear regression models were created to predict variance in methylation of seven selected CpG sites from *NR3C1* in whole blood at age 7 years, including the main predictor variable of the first principal component score of observed maternal–infant interaction quality (derived from the Thorpe Interaction Measure at 12 months of age) and covariates of cell‐type proportion, maternal financial difficulties and marital status at 8 months postnatal, child birthweight, and sex.

**Results:**

CpG site cg27122725 methylation was negatively associated with warmer, more positive maternal interaction with her infant (*β* = 0.19, *p* = .02, *q* = 0.13). In sensitivity analyses, the second highest quartile of maternal behavior (neutral, hesitant behavior) was positively associated with cg12466613 methylation. The other five CpG sites were not significantly associated with maternal–infant interaction quality.

**Conclusions:**

Narrow individual variation of maternal interaction with her infant is associated with childhood methylation of two CpG sites on *NR3C1* that may be particularly sensitive to environmental influences. Infancy may be a sensitive period for even small influences from the social–emotional environment on the epigenetic determinants of HPA‐axis function.

## 1 INTRODUCTION

Early life environments and experiences can shape child biology and health in ways that may persist throughout the lifespan and even across generations. The plasticity of human growth and development enables humans to fine‐tune body size, shape, and physiology in response to the environment, based on information from the external environment (Gluckman et al., 2005). The magnitude of plasticity is associated with the rate of growth, with many critical windows of impact occurring in periods of increased rates of growth, namely fetal, neonatal, and adolescent periods (Heim & Binder, 2012). These plastic responses can shape a person's health and functioning in adaptive and detrimental ways. How these plastic responses can form the foundation of health and disease throughout the lifespan is the focus of the research field Developmental Origins of Health and Disease (DOHaD).

A large portion of research in the DOHaD framework has analyzed the association between maternal prenatal stress and fetal development as an origin of health and disease (Entringer et al., 2015; Fox et al., 2015; Monk et al., 2012). This is a logical focus as there is a very direct influence of maternal physiology on fetal development and extremely rapid growth and development during the fetal period (Tanner, 1990). The period of infancy, however, is also a critical window as there is rapid growth and development during infancy, particularly in the brain, which is central to the stress response (Lupien et al., 2009; Tanner, 1990). Based on both rodent and human research, the hypothalamic–pituitary–adrenal (HPA) axis appears to be particularly sensitive to stress in fetal and infant development (van Bodegom et al., 2017). This timing of exposure influences how a person's physiology and phenotype is affected by stress (Goldman‐Mellor et al., 2012; Heim & Binder, 2012). Identifying the particular influences on HPA axis development is particularly important for understanding developmental plasticity more broadly, as the HPA axis works to mobilize stored energy and suppress the immune response (de Kloet et al., 2005; Ulrich‐Lai & Herman, 2009), which are also involved in determining how energy is allocated across life history traits (Kuzawa et al., 2020; Urlacher et al., 2018).

The development of the HPA axis is sensitive to social–emotional environmental cues during fetal and infant growth. Infant cortisol response to a stressor and average diurnal cortisol production have been found to be associated with maternal behavior with her infant (Galbally et al., 2019; Tarullo et al., 2017). Cortisol reactivity to a stressor may develop in infancy, as longitudinal studies have demonstrated intra‐individual variation in cortisol reactivity across infancy measured through 24 months of age (Davis & Granger, 2009; Gunnar et al., 1996; Jansen et al., 2010; Martinez‐Torteya et al., 2015; Thompson et al., 2015; Tollenaar et al., 2010) as well as the circadian secretion of cortisol (de Weerth et al., 2003; Wong et al., 2022). Extreme cases of maternal depression and anxiety have been found to negatively predict infant social engagement, regulatory behaviors, emotionality, and cortisol reactivity, with maternal sensitivity to her infant moderating some of these associations (Feldman et al., 2009).

Some of this programming of the stress response occurs through epigenetic changes (McGowan & Matthews, 2018). Epigenetic mechanisms have become particularly relevant to DOHaD research as they change during sensitive periods of development in response to the environment (Liang et al., 2011; Low et al., 2012), providing a direct mechanism by which the broader environment interacts with genotype to shape phenotype through ontological processes (Champagne, 2013; Meaney & Szyf, 2005). While there are multiple epigenetic mechanisms, including histone modification and microRNA expression, this analysis specifically explores DNA methylation. DNA methylation of *NR3C1*, the gene that encodes the glucocorticoid receptor integral to the negative feedback loop of the HPA‐axis, is associated with variation in stress response and HPA‐axis function (Oberlander et al., 2008). Early life trauma is also associated with *NR3C1* methylation variation in humans. *NR3C1* hypermethylation has occurred following parental loss (Tyrka et al., 2012), maternal prenatal exposure to war‐related stress and trauma (Mulligan et al., 2012; Perroud et al., 2014), childhood abuse (Shields et al., 2016), among other early life stress experiences (see Palma‐Gudiel et al., 2015 for a review). *NR3C1* hypermethylation is also associated with many poor health outcomes including depression (Melas et al., 2013), borderline personality disorder (Dammann et al., 2011; Steiger et al., 2013), and cancer (Kay et al., 2011; Lind et al., 2006; Nesset et al., 2014). Given the relationship between *NR3C1* and HPA axis function, the degree of *NR3C1* methylation reflects stress response physiology programming.

It is unclear whether small variations in maternal behavior, rather than extreme stress or trauma, also can shape *NR3C1* methylation in ways that persist through childhood—that is, whether the period of infancy is particularly susceptible to cues of the social–emotional environment in ways that shape *NR3C1* methylation. While some research has explored the relationship between general individual variation in maternal caregiving and infant methylation, few have explored these caregiving effects independent of adversity in early life (such as a natural disaster) on *NR3C1* methylation, and far fewer have used observational measures of maternal behavior (Provenzi et al., 2020). However, some aspects of observed maternal caregiving, particularly touch, have been associated with infant *NR3C1* methylation at 5 months of age (Conradt et al., 2019), and other research has found a mediating effect of maternal caregiving on the relationship between maternal depression and infant *NR3C1* methylation (Conradt et al., 2016; Murgatroyd et al., 2015). This suggests that maternal interaction with her infant, shaped by maternal stress and mood, can have significant impact on infant development. This may be a pathway by which maternal behavior in the postnatal period can give cues to their developing infant about the social–emotional environment, shaping HPA axis development and physiology in ways that determine responses to stress in later life.

While epigenetic programming of the HPA axis is a possible mechanism by which the external social and emotional environment can become embodied and shape stress response physiology in early life, it is not clear whether this pathway is sensitive to individual variation of maternal behavior with her child. This study seeks to test a component of this hypothesis, by evaluating whether positive, warm, engaged maternal behavior with her infant is associated with methylation of the HPA‐axis related gene, *NR3C1* at 7 years of age.

## 2 MATERIALS AND METHODS

### 2.1 Sample population

The Avon Longitudinal Study of Parents and Children (ALSPAC) is a prospective cohort study researching the growth and health of children. Detailed description of the study design is published in Boyd et al., 2013 and Fraser et al., 2013. Women residing in specified regions of Avon, UK were eligible to participate in ALSPAC if their expected delivery date was between April 1, 1991 and December 31, 1992, with an initial number of pregnancies enrolled of 14 541. The total initial sample of children in the study at 1 year of age were 13 988. An additional 913 children were enrolled in the study at the age 7 follow‐up (“Focus@7”), who met original eligibility requirements but were not enrolled in Phase 1 of the study. The ALSPAC cohort is more likely to be of higher socioeconomic status and mothers are more likely to be white than the Avon region from which they are sampled as well as the UK overall. Please note that the study Web site contains details of all the data that is available through a fully searchable data dictionary and variable search tool here: http://www.bristol.ac.uk/alspac/researchers/our-data/.

**FIGURE 1:**
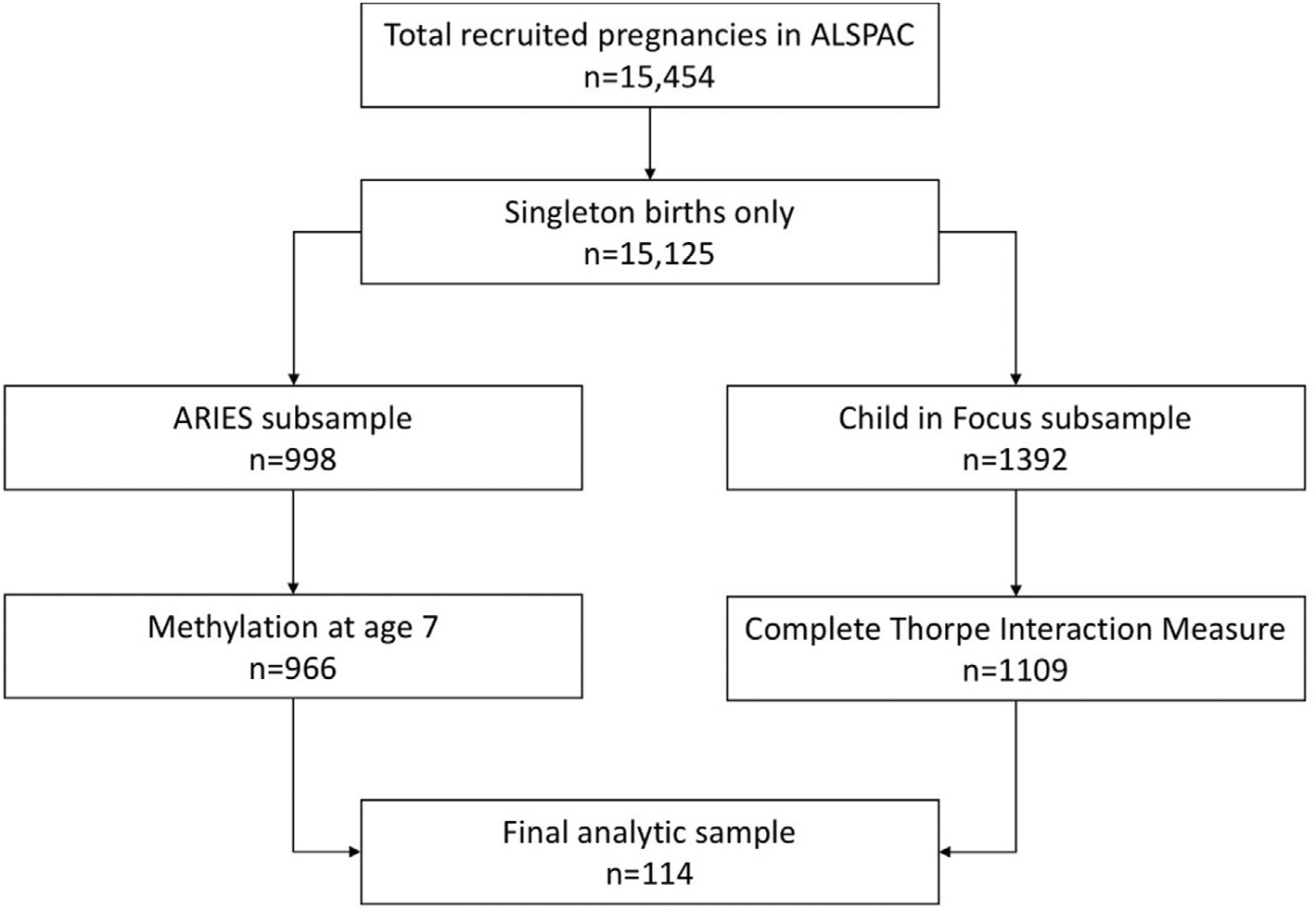
Sample flowchart

Only singleton births were included in the study. If mothers participated in the study with multiple children, only the first enrolled child was included in this analytic sample (*n* = 15 125). As part of the Accessible Resource for Integrated Epigenomic Studies (ARIES) project, a random subsample of 1018 mother–child pairs were selected for epigenome‐wide methylation analysis (Relton et al., 2015). Participants were selected based on the availability of collected DNA samples at two time points for mothers (antenatal and during offspring adolescence) and at three time points for offspring (neonatal or cord blood, 7 years of age, and 17 years of age). This analysis included only those mother–child pairs that had both fully completed the Thorpe Interaction Measure as part of the Child in Focus random 10% subsample (*n* = 1109) and had measures of child methylation at age 7 years (*n* = 966), leaving a final sample for this analysis of *n* = 114 mother–child pairs who were participants of both subsamples. We declined to impute missing values of these main variables of interest as there was over 85% missing from each variable. Sample flowchart is Figure 1.

Mothers participating in ALSPAC gave written informed consent for participation. Ethical approval for the study was obtained from the ALSPAC Ethics and Law Committee and the Local Research Ethics Committees. Consent for biological samples has been collected in accordance with the Human Tissue Act (2004), and informed consent for the use of data collected via questionnaires and clinics was obtained from participants following the recommendations of the ALSPAC Ethics and Law Committee at the time. Ethical approval for secondary data analysis of the ALSPAC dataset was granted by the Institutional Review Board at the University at Albany, State University of New York.

### 2.2 Maternal–infant interaction

The Thorpe Interaction Measure (Raine et al., 2016; Thorpe et al., 2003) was included in a clinic visit for a random subgroup of 10% of ALSPAC participants at the child's 12‐month visit. This is an observation protocol that evaluates the behavior and relationship of a mother with her child in sharing a picture book. The Thorpe Interaction Measure takes place at the research clinic where the mother is asked to share the picture book with her child in a way she would at home. One researcher evaluated all participants and these measures were video‐recorded for further review. Investigation into coding methodologies have contributed to the validity and reliability of the Thorpe Interaction Measure (Pearson et al., 2012; Puckering et al., 2014; Thomson et al., 2014). In the ALSPAC sample, this measure has been found to be sensitive to differences in behavior of depressed versus not depressed mothers, and associated with later child language outcomes. More positive maternal–infant interactions were associated with a decreased risk of oppositional/conduct disorder diagnoses at 7 years of age, and more positive interactions were associated with older maternal age and more maternal education.

To summarize maternal interaction, a principal components analysis was conducted. Specifically, variables pertaining to mother's emotional behavior with her infant were extracted to derive a principal component score. These variables include physical proximity (1 = distant, 2 = moderate, 3 = close), non‐verbal communication (1 = negative, 2 = neutral, 3 = positive), verbal communication (1 = negative, 2 = neutral, 3 = positive), control (1 = mother much more in control, 2 = mother more in control, 3 = mutual control, 4 = child more in control, 5 = child much more in control), warmth (1 = very warm, 2 = warm, 3 = neutral, 4 = cool, 5 = very cool), mother‐motivated parenting (1 = very motivated, 2 = positive, 3 = neutral, 4 = hesitant, 5 = negative), mother's reaction to child (1 = awkward, 2 = not positive, 3 = some positive reaction, 4 = positive), and mother's familiarity with the task (1 = strong, 2 = moderate, 3 = weak). These components of the mother‐infant interaction measure were standardized before principal component analysis to correct for variable differences in ranges and variance. A principal components analysis was used to summarize these scores into one usable variable, while being able to differentially weight variables and control for maternal familiarity with the task. The first principal component score was derived for all individuals and used as the predictor variable of maternal–infant interaction, as principal component one explained 49% of the variance in the sample. A higher principal component score indicated a more awkward, neutral, cooler parenting style, while a lower principal component score indicated a closer, positive, warmer parenting style.

### 2.3 *NR3C1* methylation

Peripheral blood samples were collected at a clinic visit at 7 years of age and used for epigenome‐wide methylation analyses. DNA methylation and preprocessing analyses were performed at the University of Bristol as part of the Accessible Resource for Integrated Epigenomics Studies (ARIES) project. After extraction, DNA was bisulphite‐converted using the Zymo EZ DNA MethylationTM kit (Zymo, Irvine, CA, USA). Genome‐wide methylation status of over 485 000 CpG sites was measured using the Infinium HM450 BeadChip. Arrays were scanned using an Illumina iScan and quality review conducted using GenomeStudio (version 2011.1). During data acquisition, technical covariates were collected in a purpose‐built laboratory information management system (LIMS). The LIMS also reported quality control (QC) metrics from the standard control probes on the 450 k BeadChip (Relton et al., 2015). Samples failing quality control (average probe detection *p*‐value ≥.01) were excluded from further analyses and scheduled for repeat assay.

Methylation levels were measured as beta (*β*) values as defined by the ratio of methylated probes intensities and total probes intensities at a CpG locus. Beta values were pre‐processed in R (version 3.0.1) according to the subset quantile normalization approach described by Touleimat & Tost, 2012. Probes with *p*‐values ≥.01 for more than 5% subjects were excluded and extreme outliers (identified as methylation values greater than Q3 + 3IQR or less than Q1‐3IQR) were removed from analyses. Retaining only CpG loci on the autosomes and excluding CpG loci reported to contain single nucleotide polymorphisms, small insertions and deletions, repetitive DNA, and regions with reduced genomic complexity (Naeem et al., 2014), a total of 285 994 CpG loci remained for analyses at childhood (7 years of age).

This analysis focused on the 39 different CpG sites in the 450 k array remaining after QC that are a part of the *NR3C1* gene. To reduce the dimensionality of *NR3C1* and prevent type 1 error, we examined the methylation variability across the CpGs and excluded sites with little to no variation (and thus would not be informative in subsequent analyses). Specifically, preliminary analyses on variance of methylation across these 39 sites demonstrated that the vast majority of CpG had very low variation across individuals (standard deviations <0.05), making it unlikely to be able to identify causes of variation at these sites. Seven CpG sites with standard deviations ≥0.05 were selected for further analysis. Sites with low methylation variation were excluded in order to avoid unnecessary testing and overly stringent corrections for multiple testing (Essex et al., 2011; Paquette et al., 2016).

### 2.4 Covariates

Demographic variables were selected based on their possible association with either maternal–infant interaction quality or with *NR3C1* methylation in childhood. These variables considered are listed in Table 1. Maternal financial difficulties and opinion of neighborhood self‐reported at 8 months postnatal were selected to approximate socioeconomic status close to the time of the maternal–infant interaction measure. Three subjects who had data for both the main predictor and outcome variables were missing data for some covariates, including infant birthweight (one missing), mother's marital status at 8 months postnatal (one missing), financial difficulties score at 8 months postnatal (two missing), maternal partner at 8 months postnatal (one missing), and maternal opinion of neighborhood at 8 months (one missing). These missing values were imputed using predictive mean matching with 50 iterations using the *mice* package in R (van Buuren & Groothuis‐Oudshoorn, 2011). Table 1 displays univariate statistics before imputation. Spearman correlation coefficients were evaluated for all possible covariate variables along with maternal–infant interaction composite and domain scores, and methylation of the selected CpG sites. *Q*‐values for bivariate correlations were generated based on the false discovery rate (FDR). Variables correlated (*p* < .05) with either the maternal–infant interaction principal component one score or any of the selected CpG sites methylation values were included as covariates in the following regression models, along with infant sex and cell‐type composition.

**TABLE 1:** Sample descriptive statistics compared to total ALSPAC sample

	*N* (%) or mean ± *SD* Analytic sample (*n* = 114)	*N* (%) or mean ± *SD* Total ALSPAC sample (*n* = 15 125)
Sex (Female)	59 (51.8%)	7152 (48.9%)^a^
Maternal age at delivery, years*	29.5 ± 4.4	28.0 ± 5.0^b^
Gestational age at birth, weeks*	39.7 ± 1.4	38.5 ± 5.5^c^
Birthweight, g*	3553.7 ± 424.4	3405.5 ± 559.9^d^
Delivery method, Vaginal C‐section*	109 (95.6%) 5 (4.4%)	6634 (82.7%) 1392 (17.3%)^e^
Maternal ethnic group, white	113 (99.1%)	11 748 (97.4%)^f^
Mother married at 8 months postnatal*	100 (88.5%)	8732 (79.4%)^g^
Mother has partner at 8 months postnatal	111 (98.2%)	10 507 (95.5%)^h^
Maternal financial difficulties score at 8 months postnatal*	2.2 ± 2.9	3.2 ± 3.6^i^
Maternal opinion of neighborhood at 8 months postnatal, very good or good	108 (94.7%)	10 139 (92.1%)^j^
Mother‐infant interaction score	−0.16 ± 2.22	−0.01 ± 1.98^k^
cg13648501	0.19 ± 0.05	0.17 ± 0.05^l^
cg06952416	0.21 ± 0.05	0.19 ± 0.05^l^
cg07733851	0.30 ± 0.07	0.29 ± 0.07^l^
cg27122725	0.32 ± 0.07	0.30 ± 0.07^l^
cg18998365	0.51 ± 0.06	0.53 ± 0.06^l^
cg12466613	0.60 ± 0.09	0.64 ± 0.09^l^
cg19457823	0.64 ± 0.09	0.68 ± 0.09^l^

* *p* < .05 test of differences between analytic sample and all other ALSPAC data.

^a^ *n* = 14 626 due to missing values.

^b^ *n* = 13 697 due to missing values.

^c^ : n = 4216 due to missing values.

^d^ *n* = 13 525 due to missing values.

^e^ *n* = 8026 due to missing values.

^f^ *n* = 12 062 due to missing values.

^g^ *n* = 10 991 due to missing values.

^h^ *n* = 10 997 due to missing values.

^i^ *n* = 10 985 due to missing values.

^j^ *n* = 11 006 due to missing values.

^k^ *n* = 1109 due to missing values.

^l^ *n* = 970 due to missing values.

A variable measuring the cell type of the tissue sample used in methylation analysis was derived through a principal component analysis (Gervin et al., 2016). Relative proportions of B, CD4 T, CD8 T, granulocytes, monocytes, and NK cells were included in a principal component analysis. The first principal component was determined to explain a sufficient amount of variation in cell type (45%), so scores of principal component one were derived for every methylation sample (Koestler et al., 2013).

### 2.5 Statistical analyses

Analyses were conducted using RStudio (R Core Team, 2020). The dependent variable, methylation, is distributed on a scale of 0 (fully unmethylated) to 1 (fully methylated). Linear regression models were created to test the relationship between predictor variables and methylation. Seven models on each of the selected CpG sites were created using the maternal–infant interaction principal component score as the predictor variable and covariates of cell‐type principal component score, child sex, birthweight, maternal reported financial difficulties score at 8 months postnatal, and maternal marital status at 8 months postnatal. Additional sensitivity analyses were conducted by creating quartiles of maternal–infant interaction principal component scores, and used in linear regression models along with infant sex, cell‐type composition, birthweight, maternal reported financial difficulties score at 8 months postnatal, and maternal marital status at 8 months postnatal to predict variance in methylation of the selected CpG sites. In order to account for multiple testing, *q*‐values based on the false discovery rate (FDR) were calculated from each model's *p*‐value of maternal–infant interaction score. Results were considered significant if *q* < 0.25, indicating medium confidence of significant results (Lam et al., 2012).

## 3 RESULTS

### 3.1 Descriptive results

This analytic sample was significantly different from the overall ALSPAC study population in a few demographic domains (Table 1). Mothers in this sample were older at delivery, had longer gestations and babies of higher birthweight, and were more likely to have a spontaneous, unassisted vaginal delivery and less likely to have c‐section deliveries. Mothers also reported fewer financial difficulties on average at 8 months postnatal compared to the rest of the ALSPAC population. Similar to the overall ALSPAC population, this sample of mothers was overwhelmingly of white ethnicity, mothers were mostly married or had a partner at 8 months postnatal, and had a positive opinion of their neighborhood.

The principal component 1 score describing maternal interaction with her infant (Table 2) was primarily driven by more awkward or not positive reactions to her child, more hesitant mother‐motivated parenting, weak maternal familiarity with the task, more negative verbal communication, cooler or neutral maternal behavior, and more negative non‐verbal communication. Physical proximity and control were not large contributors to this score. Overall, higher values on this score represented cooler, less positive interactions from mother to her child.

**TABLE 2:** Distribution of Thorpe Interaction Measure domain scores and contribution to principal component 1 scores

Measure (score range)	Mean ± *SD*	Principal component 1 loadings
Physical proximity (1–3, higher values are closer proximity)	2.9 ± 0.34	−0.054
Non‐verbal communication (1–3, higher values being positive)	2.6 ± 0.57	−0.364
Verbal communication (1–3, higher values being positive)	2.7 ± 0.51	−0.393
Control (1–5, higher values being child more in control than mother)	2.4 ± 0.83	−0.032
Warmth (1–5, higher values being very cool)	2.0 ± 0.66	0.370
Mother‐motivated parenting (1–5, higher values being hesitant or negative parenting)	2.2 ± 0.82	0.439
Mother's reaction to child (1–4, higher values being positive reactions)	3.0 ± 0.77	−0.458
Mother's familiarity with task (1–3, higher values being weak familiarity)	1.9 ± 0.58	0.412

Methylation across CpG sites were on average low (Table 1) to moderately methylated (e.g., cg19457823 mean = 0.64). As these seven CpG sites were selected for their higher degree of variance in methylation across individuals, mean methylation of these CpG sites are more likely to be within mid‐ranges than CpG sites on *NR3C1* not selected for analysis (e.g., cg18019515 mean = 0.01).

In bivariate Spearman correlations, mother's opinion of neighborhood at 8 months postnatal was negatively associated with physical proximity (*r* = −0.19, *p* < .05, *q* = 0.21) meaning a more positive opinion of her neighborhood was associated with more distant maternal proximity with her child. C‐section mode of delivery was positively correlated with methylation at site cg19457823 (*r* = 0.21, *p* < .05, *q* = 0.13). Maternal age at delivery was negatively correlated with control (*r* = −0.20, *p* < .05, *q* = 0.17), with younger mothers being associated with more child rather than maternal control in the task. Maternal marital status at 8 months postnatal was positively correlated with maternal familiarity with the task (*r* = 0.12, *p* < .05, *q* < 0.05), and methylation at sites cg08818984 (*r* = 0.19, *p* < .05, *q* = 0.20), cg18998365 (*r* = −0.20, *p* < .05, *q* = 0.16), cg12466613 (*r* = −0.20, *p* < .05, *q* = 0.17) and with methylation at site cg19457823 (*r* = −0.19, *p* < .05, *q* = 0.20). Birthweight was positively correlated with methylation at site cg07733851 (*r* = 0.19, *p* < .05, *q* = 0.21) and mother's familiarity with task (*r* = −0.19, *p* < .05, *q* = 0.21). Maternal financial difficulties at 8 months postnatal were correlated with methylation at sites cg06952416 (*r* = 0.23, *p* < .05, *q* = 0.06) and cg06613263 (*r* = −0.19, *p* < .05, *q* = 0.20), indicating greater methylation at site cg06952416 with more financial difficulties, and lower methylation at site cg06613263. No covariates were significantly correlated with maternal–infant interaction principal component one score.

### 3.2 Maternal–infant interaction and CpG methylation

Table 3 lists the associations between domains of maternal–infant interaction and *NR3C1* CpG specific methylation. The majority of CpG sites were not significantly associated with maternal–infant interaction scores. CpG site cg27122725 methylation was significantly predicted by maternal–infant interaction with a *β* estimate of 0.214 (*SE* = 0.090; *p* = 0.02), controlling for cell type composition, maternal marital status at 8 months postnatal, maternal financial difficulties at 8 months postnatal, child birthweight, and sex. The overall model adjusted *R*‐squared value was 0.13, indicating that this model explains 13% of the variance in methylation at cg27122725. It is not likely that this significant relationship is a false positive, given the *q*‐value of 0.13, below the FDR threshold of *q* < 0.25. This means that one standard deviation increase in the mother‐infant interaction score, toward cooler, more neutral maternal behavior, is associated with a 0.21 standard deviation increase in methylation, or an increase of 1.5% methylation.

**TABLE 3:** Linear regression models predicting CpG methylation at each site at age 7 years

CpG site	Location on NR3C1, relation to CpG island (genomic location, bp)	*β* of maternal–infant interaction (model adjusted *R*‐squared)	*p* (*q*)
cg13648501	5′ UTR, S. Shore (5:143405693–143 405 742)	0.008 (0.07)	0.93 (0.93)
cg06952416	5′ UTR, N. Shore (5:143402123–143 402 172)	0.043 (0.07)	0.64 (0.93)
cg07733851	5′ UTR, N. Shore (5:143401885–143 401 934)	−0.030 (<0.01)	0.76 (0.93)
**cg27122725**	5′ UTR, N. Shore (5:143402158–143 402 207)	**0.214 (0.13)**	**0.02 (0.13)**
cg18998365	5′ UTR, N. Shore (5:143401967–143 402 016)	−0.075 (0.01)	0.43 (0.93)
cg12466613	TSS1500 (5:143435904–143 435 953)	0.014 (0.12)	0.88 (0.93)
cg19457823	Body (5:143313348–143 313 397)	−0.025 (0.14)	0.78 (0.93)

*Note*: Models include maternal–infant interaction variable, cell‐type heterogeneity, maternal financial difficulties score at 8 months postnatal, maternal marital status at 8 months postnatal (married/not married), child birthweight, and sex. Bold indicates maternal–infant interaction significantly predicts methylation at this site at *p* < .05 and *q* < 0.25. Location on genome from Ensembl (GRCh38.p13). 5′ UTR = five prime untranslated region. TSS1500 = 200–1500 bases upstream of the transcription start site.

**FIGURE 2:**
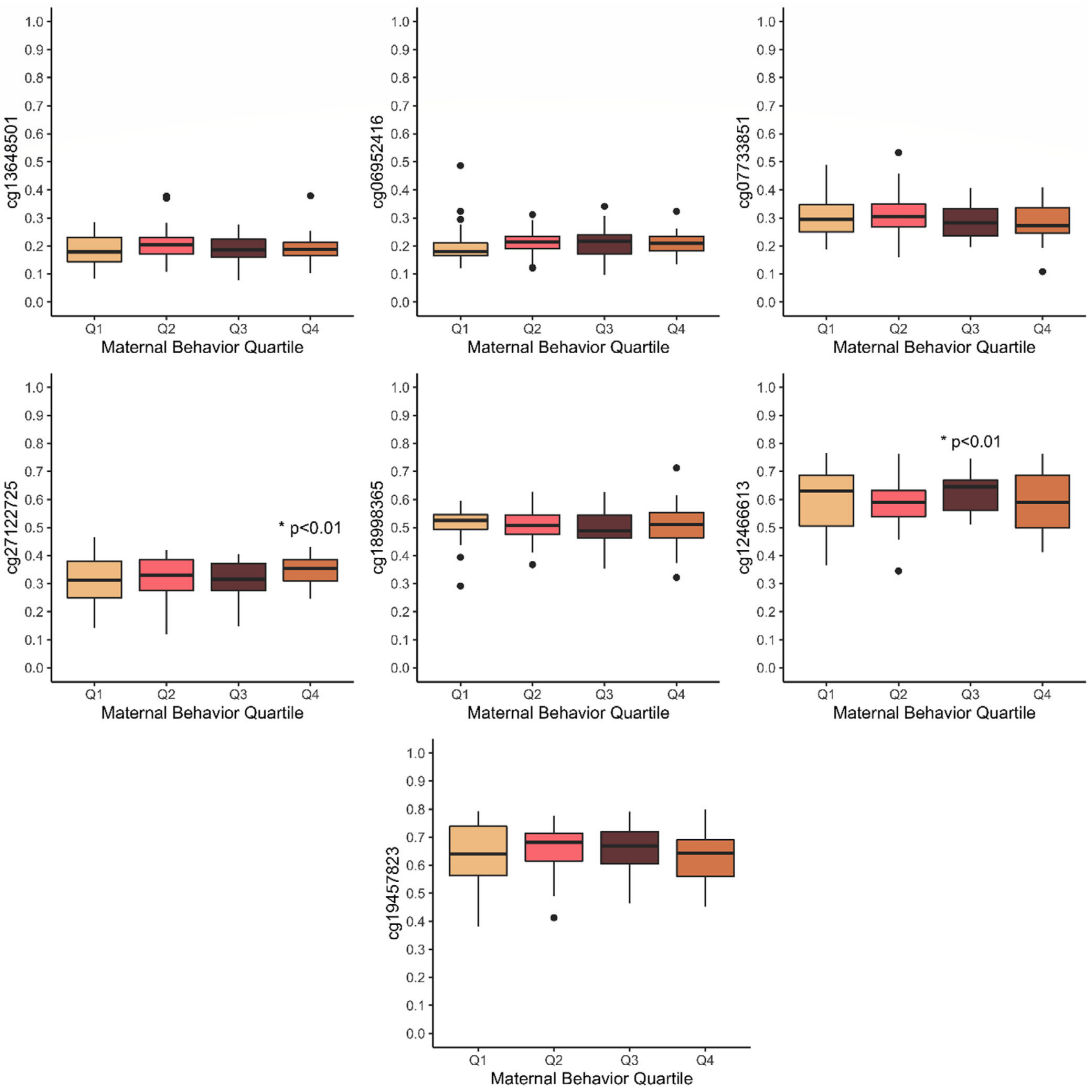
Maternal behavior scores by quartile and methylation of CpG sites. *Indicates *p* < .05 significance of quartile in linear regressions including cell‐type heterogeneity, maternal marital status at 8 months postnatal, maternal financial difficulties at 8 months postnatal, child birthweight, and sex

Figure 2 shows associations between quartiles of the mother‐infant interaction principal component one scores in relation to *NR3C1* methylation at each CpG site. Sensitivity analyses were conducted with quartiles of mother‐infant interaction scores compared to methylation of each CpG site. Linear regression models including cell‐type heterogeneity score, maternal marital status at 8 months postnatal, maternal financial difficulties at 8 months postnatal, child birthweight, and sex showed that maternal–infant interaction quartiles was significantly associated with CpG methylation in cg27122725 and cg12466613. The second highest quartile of maternal–infant interaction scores significantly positively predicted cg12466613 methylation (*β* = 0.21, *p* = .02, adjusted *R*‐squared = 0.17). The highest quartile of maternal–infant interaction scores significantly positively predicted cg27122725 methylation (*β* = 0.23, *p* = .01, adjusted *R*‐squared = 0.14).

## 4 DISCUSSION

These results indicate with medium confidence that cooler, more awkward maternal behavior with her infant at 12 months of age was associated with increased methylation at CpG site cg27122725 at age 7 years, with the strongest association of increased methylation with the highest quartile of maternal cooler, less positive behavior. Increased methylation of cg12466613 was associated with only the second highest quartile of more neutral maternal behavior. Most CpG sites were not associated with maternal behavior with her infant, indicating that influences on *NR3C1* methylation may only occur on specific CpG sites, rather than widespread across the gene, similar to previous studies (Palma‐Gudiel et al., 2015). This research suggests that small, relatively mundane variation in maternal behavior may be associated with small variation in *NR3C1* specific CpG site methylation at age 7 years, particularly among the CpG site cg27122725, but not with most CpG sites. This is a particularly important finding for understanding how HPA‐axis‐related gene expression may be a developmentally plastic pathway that is sensitive to maternal behavior in early life.

It is important to note that maternal interaction with her infant was largely rated as positive or neutral in this sample. Thus, methylation of these CpG sites is in association with a relatively narrow range of individual variation in maternal behavior, rather than a response to adverse conditions or “negative” caregiving behavior. The small effect size association with only a few CpG sites suggests that these small methylation differences may simply reflect phenotypic variation in response to common variation in the socio‐emotional environment. Furthermore, across all studies of maternal–infant interactions, it is important to emphasize that maternal caregiving behavior is also determined by sociocultural factors, such as stress from lack of resources or support, exposure to racism, environmental unpredictability, maternal depression, and poverty (Berry et al., 2021; Herba et al., 2016; McCurdy, 2005; O'Dea & Marcelo, 2022; Rosenblum & Andrews, 1994). It is necessary to consider the social determinants of maternal mental health and early child development, such as poverty and poor societal and sanitation infrastructure, in order to most effectively and jointly improve well‐being among mothers and infants (Pitchik et al., 2020).

These results complement recent findings that individual variation in maternal caregiving behavior may be related to *NR3C1* methylation at specific CpG sites. Previously, *NR3C1* methylation changes at select CpG sites have been documented following exposure to severe psychological and emotional stress, trauma, and neglect (Palma‐Gudiel et al., 2015), as well as exposure to maternal psychological stress and depression, though many of the results are discordant and highly variable in timing of outcome, exposure, and methylation tissue type (Berretta et al., 2021). Specifically, while cg13648501 was found to be differentially methylated in whole blood in childhood (mean age 10 years) by exposure to maltreatment, cg27122725 and cg12466613 were not significantly associated with maltreatment (Weder et al., 2014), contrary to the findings of this analysis. Other recent research has sought to test in humans the groundbreaking findings of maternal caregiving behaviors being associated with changes in *NR3C1* methylation of select CpG sites in mice (Weaver et al., 2004). *NR3C1* methylation variation has been found in response to common and protective maternal caregiving behaviors (Provenzi et al., 2020), rather than solely adverse experiences. Notably, maternal positive caregiving behaviors (sensitivity, responsiveness, and physical stroking) have been associated with reduced *NR3C1* methylation at specific CpG sites in female infants at 5 months of age (Conradt et al., 2019) and in infants of depressed mothers at 5 weeks and 4 months of age (Conradt et al., 2016; Murgatroyd et al., 2015). This research found similar patterns of positive caregiving behaviors being associated with lower *NR3C1* methylation at two select CpG sites (cg27122725 and cg12466613), adding to these findings a wider variety of types of caregiving behavior (maternal verbal and non‐verbal communication, reaction to child, overall warmth, and mother‐motivated parenting) that are associated with *NR3C1* methylation. Alongside previous research, this suggests that positive maternal caregiving practices are largely associated with decreased site‐specific *NR3C1* methylation. Comparisons of CpG‐specific effects across studies are challenging, however, as many different sites are used in different studies—this is extensively detailed and described by Palma‐Gudiel et al. (2015) and Berretta et al. (2021). Future research should aim to include a wide range of early life stress and positive caregiving experiences, along with multiple tissue‐type samples, to more precisely describe this relationship.

The majority of maternal behavior in this sample is evaluated as warm and positive. The ALSPAC population is more financially and nutritionally stable than the average human sample (Fraser et al., 2013) with this analytic sample more financially stable than the total ALSPAC population. A lack of increased *NR3C1* methylation could be an appropriate biological state in a nurturing, positive, and engaged socioemotional environment. Within a developmental plasticity of stress framework, the stress response should develop with an increase in sensitivity and reactivity in an environment of increased threat or stress (Nettle & Bateson, 2015). This has been demonstrated in many cases, with increased cortisol reactivity and sensitivity following early life stress or trauma (Bair‐Merritt et al., 2015; Luecken et al., 2013; Thayer & Kuzawa, 2014) though other studies have found decreased cortisol reactivity or no associations (de Rooij, 2013; Goldman‐Mellor et al., 2012; Ouellet‐Morin et al., 2011; Tollenaar et al., 2010). Increased methylation of cg27122725 in association only with the most neutral, hesitant, and less warm maternal interaction with her infant indicates that a lack of increased *NR3C1* methylation may be an appropriate biological state in an overall positive maternal caregiving environment. It is also notable that the observed effect sizes of these associations were small, with an estimated increase of 1.5% cg27122725 methylation with each additional standard deviation in maternal–infant interaction score, or 5.0% increased methylation for the most cool, distant maternal–infant interaction score measured in this sample. This is a small amount of variation in a CpG site that ranged from 11.9% to 46.6% methylation in this sample. However, similar effect sizes in CpG site methylation measured in blood, including at cg27122725, have been associated with variation in cortisol in adults (Chatzittofis et al., 2021), indicating that even small variation in specific CpG sites may contribute to biological variation in HPA axis function.

These results demonstrating an association between maternal caregiving behavior in infancy with childhood methylation at specific CpG sites are similar to previous findings that maternal caregiving behavior in infancy was associated with epigenome‐wide methylation in childhood 4–5 years later, though notably not in *NR3C1* (Moore et al., 2017). Infant attachment styles have also similarly been associated with epigenome‐wide DNA methylation in childhood (mean age 7 years; Garg et al., 2018). However, it is possible that the associations between maternal behavior in infancy and childhood *NR3C1* methylation are not exclusively attributable to experiences in infancy. During the 6 years between the maternal–infant interaction measure and the measure of *NR3C1* methylation, children may be exposed to a variety of experiences and environments that may also affect *NR3C1* methylation. The results presented here account for some measures of such potential confounders (socioeconomic factors), but other unmeasured confounders or explanations for these associations are possible. Maternal behavior with her child may change over time. For example, in one study of parenting behaviors within the ALSPAC sample, maternal engagement with her infant increased from 6 to 38 months, and then remained stable through 42 months (Gutman & Feinstein, 2010). It is possible that maternal consistency in behavior may be more important than critical periods in infant development (Landry et al., 2001). Thus maternal–infant interaction may reflect overall parenting trajectories through childhood that are associated with child methylation of *NR3C1*, rather than exclusively a sensitive period for social–emotional factors in infancy. Though infancy is certainly a developmentally sensitive period, further research is needed to clarify how persistent methylation changes in infancy are, particularly whether they do in fact persist through childhood, as this study was not able to directly test.

While maternal–infant interaction has been associated with infant growth and development (Albers et al., 2008; Holdsworth & Schell, 2017), these results suggest that *NR3C1* methylation may not be the main biological pathway for these associations. Programming of the HPA axis in general has been proposed to be mechanism by which energy is allocated across life history traits including growth (Worthman & Kuzara, 2005). Much research on HPA axis programming has focused on *NR3C1* methylation, but as there are multiple genes involved in regulating HPA axis activity (Arnett et al., 2016), it is not possible to derive clear conclusions about the HPA axis programming from only *NR3C1* methylation. Furthermore, HPA axis function may be directed in ways that are not epigenetic (Sheng et al., 2021). Significantly more research is needed to identify the epigenetic and non‐epigenetic mechanisms of developmental plasticity in response to the social, emotional, psychological environment in infancy, particularly exploring the interaction and expression of multiple HPA‐axis related genes.

While analyses of effects on aggregate gene methylation such as average methylation or principal component scores are useful, these results also point to a need to review associations at specific CpG sites as well. Exploration of individual CpG sites serves multiple functions: (1) allows for clearer cross‐sample comparisons, in order to identify variation in these exposure‐methylation patterns across populations and environments, and (2) allows for more biologically meaningful conclusions. The function of a gene is not uniform across all CpG sites. In particular, cg27122725 is located in the north shore region of a CpG island located at the proximal promoter region of *NR3C1*. Much research on *NR3C1* methylation has focused on this CpG island within the proximal promoter region exon 1F and 1F promoter (Oberlander et al., 2008). CpG islands are small regions of unmethylated DNA in the promoters of a few human genes that are frequently assumed to have the greatest functional significance (Deaton et al., 2011; Deaton & Bird, 2011; Takai & Jones, 2002). However, as Shields et al. (2016) have argued, CpG island shores are also functionally significant and appear to be sensitive to environmental exposures. CpG island shores are genomic regions located within 2000 bp of a CpG island, and are enriched with functional methylation sites that control gene expression (Doi et al., 2009; Irizarry et al., 2009; Qu et al., 2015; Wilson et al., 2014). The significant relationship with this CpG site located on the shore region upstream of the CpG island, when the other regions of the island do not show significant relationships, may indicate a greater degree of environmental sensitivity in shore regions. Furthermore, previous research found that cg27122725 methylation has been positively correlated with overall NR3C1 gene expression in whole blood as well as HPA‐axis reactivity in adults (Chatzittofis et al., 2021). More region‐specific analyses are required to test how environmental effects on methylation may vary across the epigenome, and their functional significance.

The demographics of this analytic sample limits generalizability of these results. Gestational age at birth and birthweight were significantly higher and heavier in this analytic sample than the ALSPAC sample as a whole, likely attributable to inclusion of all pregnancy outcomes in the ALSPAC sample compared to the inclusion criteria of this analytic sample of the child having aged to 7 years old. Women in the analytic sample were also more likely to have unassisted, spontaneous deliveries and to be older at the time of delivery compared to the ALSPAC sample overall. The ALSPAC sample is in general more white and more socioeconomically advantaged than the larger English population (Fraser et al., 2013), and this was reflected in this analytic sample which reported 99% white ethnic group of mothers, and 95% very good or good maternal opinion of her neighborhood. This analytic sample was even more economically advantaged than the overall ALSPAC sample, with significantly fewer maternal reported financial difficulties.

There are some limitations to this study that may bias the results toward null findings. With a sample size of *n* = 114, there was 80% power to detect an effect size of a single regression coefficient in a model with six predictor variables of *f* ^2^ = 0.070 or *β* = 0.26. Thus, lack of significant associations with the other CpG sites may be due to inadequate power, rather than a true lack of significant associations (Type II error). Significantly, methylation at age 7 years is measured in peripheral blood samples. Methylation patterns may vary across tissue‐type (Armstrong et al., 2014). Previous researchers have argued that analyses of genes relating to brain function, including *NR3C1*, is most appropriately measured in saliva samples (Smith et al., 2015), though recent research has found stability of methylation levels using the Illumina 450 k array that is used in this analysis across tissue type (Forest et al., 2018). It is possible that the small differences in methylation observed in this study are due to measuring methylation in this tissue type. However, as many other studies of early life stress have assessed *NR3C1* in whole blood samples, these results can be confidently compared to other whole blood sample studies (Edgar et al., 2017). The Illumina 450 K array measures selected 450 000 CpG sites across the epigenome, excluding many CpG sites. This means that while results can be compared to numerous other studies using the Illumina 450 K array, they cannot replicate findings from gene‐specific analyses that include other CpG sites, such as the foundational study from Weaver et al. (2004). Additionally, there is not a large degree of variation in the measure of maternal–infant interaction, with the majority of mothers displaying overall positive and warm interactions with their child. This restricted range may be unable to identify a relationship between maternal–infant interaction and *NR3C1* methylation, meaning that there may be more significant effects of maternal–infant interaction, particularly those arising from severely negative, neglectful, or abusive maternal behavior, on *NR3C1* methylation than were detected in this study. Lastly, this measure of maternal–infant interaction is limited in its ability to characterize maternal–infant interactions throughout the child's daily life. Though this measure is preferable to many alternatives as it involves direct, controlled, and comparable observation, it certainly cannot capture mothers' behaviors when they are not observed, or the active decisions mothers may make about the situations they create to engage and connect to their child. It also cannot capture the many other factors that can shape a child's psychosocial and emotional environment, such as other adult family members and fathers, peer siblings and friends, and neighborhood, day care, and school.

These limitations notwithstanding, this study has a number of strengths. This study uses observed, rather than self‐reported, maternal behavior with her infant which results in stronger validity of this measure as a reflection of the ongoing maternal–infant relationship. This measure is also assessed at a critical period in infant social–emotional development, as HPA axis reactivity and diurnal rhythm appear to change from birth to 18 months of age (Gunnar & Quevedo, 2007; Gunnar et al., 1996; Howland et al., 2017; Lewis & Ramsay, 1995), meaning that 12 months falls within a sensitive period for programming of HPA axis development. Additionally, given the longitudinal nature of this study, this work is able to demonstrate the temporal relationship between maternal–infant interaction quality and childhood *NR3C1* methylation, supporting the hypothesis that maternal behavior in infancy can epigenetically program the HPA axis in ways that persist through 7 years of age.

## 5 CONCLUSION

This study has demonstrated that variation in a common component of an infant's social–emotional environment—maternal behavior with her child—is linearly, inversely associated with methylation at *NR3C1* CpG sites cg27122725 and may be associated with cg12466613 methylation at 7 years of age. These sites, especially cg27122725, may be particularly sensitive to the early life social–emotional environment. This study provides preliminary support for the hypothesis that infancy may be a particularly sensitive period for the development of social–emotional systems such as the HPA axis, through epigenetic programming of HPA‐axis genes measured in childhood. There are extensive avenues for future research including replication of these results in other social–emotional environments using naturalistic observations, identifying the effects on and impacts of the methylation of other HPA‐axis genes such as *FKBP5*, and specifying which aspects of the social–emotional environment have an impact on *NR3C1* methylation with particular focus on severity and chronicity.

## AUTHOR CONTRIBUTIONS

**Elizabeth A. Holdsworth:** Conceptualization; data curation; formal analysis; funding acquisition; investigation; methodology; project administration; resources; software; supervision; validation; visualization; writing – original draft; writing – review and editing. **Lawrence M. Schell:** Funding acquisition; project administration; supervision; writing – review and editing. **Allison A. Appleton:** Writing – review and editing.

## CONFLICT OF INTEREST STATEMENT

The authors declare no conflicts of interest.

## Data Availability

The data that support the findings of this study are available from University of Bristol. Restrictions apply to the availability of these data, which were used under license for this study. Data are available from http://www.bristol.ac.uk/alspac/ with the permission of University of Bristol.
